# Protective or Deleterious Role of Scavenger Receptors SR-A and CD36 on Host Resistance to *Staphylococcus aureus* Depends on the Site of Infection

**DOI:** 10.1371/journal.pone.0087927

**Published:** 2014-01-31

**Authors:** Charlène Blanchet, Gregory Jouvion, Catherine Fitting, Jean-Marc Cavaillon, Minou Adib-Conquy

**Affiliations:** 1 Institut Pasteur, Cytokines & Inflammation, Département Infection et Epidemiologie, Paris, France; 2 Institut Pasteur, Unité d'Histopathologie humaine et modèles animaux, Département Infection et Epidemiologie, Paris, France; University of California Merced, United States of America

## Abstract

*Staphylococcus aureus* is a major human opportunistic pathogen responsible for a broad spectrum of infections ranging from benign skin infection to more severe life threatening disorders (e.g. pneumonia, sepsis), particularly in intensive care patients. Scavenger receptors (SR-A and CD36) are known to be involved in *S. aureus* recognition by immune cells in addition to MARCO, TLR2, NOD2 and α5β1 integrin. In the present study, we further deciphered the contribution of SR-A and CD36 scavenger receptors in the control of infection of mice by *S. aureus*. Using double SR-A/CD36 knockout mice (S/C-KO) and *S. aureus* strain HG001, a clinically relevant non-mutagenized strain, we showed that the absence of these two scavenger receptors was protective in peritoneal infection. In contrast, the deletion of these two receptors was detrimental in pulmonary infection following intranasal instillation. For pulmonary infection, susceptible mice (S/C-KO) had more colony-forming units (CFU) in their broncho-alveolar lavages fluids, associated with increased recruitment of macrophages and neutrophils. For peritoneal infection, susceptible mice (wild-type) had more CFU in their blood, but recruited less macrophages and neutrophils in the peritoneal cavity than resistant mice. Exacerbated cytokine levels were often observed in the susceptible mice in the infected compartment as well as in the plasma. The exception was the enhanced compartmentalized expression of IL-1β for the resistant mice (S/C-KO) after peritoneal infection. A similar mirrored susceptibility to *S. aureus* infection was also observed for MARCO and TLR2. *Marco and tlr2* -/- mice were more resistant to peritoneal infection but more susceptible to pulmonary infection than wild type mice. In conclusion, our results show that innate immune receptors can play distinct and opposite roles depending on the site of infection. Their presence is protective for local pulmonary infection, whereas it becomes detrimental in the peritoneal infection.

## Introduction

*Staphylococcus aureus* is a major human opportunistic pathogen responsible for a broad spectrum of infections ranging from food poisoning and superficial skin abscesses to more serious diseases such as pneumonia, meningitis, osteomyelitis, endocarditis, septicemia or toxic shock syndrome [1]. Systemic diseases caused by *S. aureus* reflect its impressive capacity to subvert the defenses of the human innate immune system [2]. The unique adaptive potential displayed by *S. aureus* has made it one of the major causes of nosocomial infections nowadays. It is also the most commonly found bacterium in patients admitted in intensive care units (ICU), representing 30% of infections and including 14% of methicillin-resistant strains (MRSA) [3].

Several innate immune receptors are involved in the detection of *S. aureus*. The membrane Toll-like receptor 2 (TLR2) is involved in the detection of lipoteichoic acid (LTA), lipoproteins and eventually peptidoglycan [4]–[8]. The cytosolic nucleotide oligomerization domain 2 (NOD2) protein permits the detection of bacterial peptidoglycan, via its minimal muramyl dipeptide moiety [9], [10]. Host cell integrin α5β1 is involved in the host response to *S. aureus* [11]–[13] as well as other receptors belonging to the family of scavenger receptors, such as SR-A, MARCO (class A) and CD36 (class B) [14]–[20].

The relative importance of these various innate immune receptors in the resistance to *S. aureus* infection remains controversial. Studies published so far are difficult to compare, because they used different strains, more or less virulent, or clinical isolates. *S. aureus* expresses multiple virulence factors, including exotoxins, LTA, adhesins, hemolysins, cytotoxins, staphylokinase, plasminogen activating factor and capsular polysaccharide that may modulate its pathogenicity. In addition, the various *in vivo* models also differ in terms of inoculation route. As an example, the absence of TLR2 had minimal impact, when *S. aureus* was administered in the peritoneum or subcutaneously [21], [22]. In contrast, other studies using different bacterial strains and intravenous infection found *tlr2*-/- mice more susceptible to *S. aureus* [23], [24]. Similarly for NOD2, recent studies report very different results. In one model of peritoneal infection, *nod2*-/- mice were highly susceptible to *S. aureus* [25], whereas this was not seen in a skin or a lung model of infection [26], [27]. These contradictions may be due to the use of *S. aureus* strains of different virulence, different bacterial loads, and different route of infection.

In this study, we focused on the role of SR-A and CD36 in host resistance to *S. aureus* infection, because few data are available on knockout mice for these receptors *in vivo* with this pathogen. Both scavenger receptors are expressed by peritoneal [28] and alveolar macrophages [29]. They often share similar activities [29]–[30] and silencing of one favors the upregulation of the other [31]. These observations suggest that there might be some redundancy with these two receptors, therefore we used double KO mice.

Most of the studies showing their role in the detection of *S. aureus* were performed *in vitro* and/or with blocking antibodies. *In vivo* infection (cutaneous or systemic) was only performed for CD36 [17], [23]. Because *S. aureus* is present for 68% of the reported cases in the lungs and 22% in the abdomen of infected ICU patients [3], we chose to compare pulmonary *versus* peritoneal infection. In the light of the heterogeneity of the strains used so far to study *S. aureus*-host interaction, we decided to use a well-characterized strain (HG001) [32], derived from the NCTC 8325 strain, for which the complete genome sequence is available [33].

After infection, we did a survey in terms of mortality, bacterial load locally and in the blood, of neutrophil and macrophage recruitment, induction of cytokines and chemokines and histology. Our data show that the outcome of wild type (C57BL/6J) and SR-A/CD36 deficient mice (S/C-KO) depends on the site of infection, with mirrored results after intra-nasal or intra-peritoneal infection. SR-A and CD36 expression is protective in pulmonary, but deleterious in peritoneal infection.

## Materials and Methods

### Mice

The studies were performed with 8 to 12 week-old male C57BL/6(J) (B6J) purchased from Janvier (Le Genest-St.-Isle, France), and knockout mice bread in our animal facilities and all in a C57BL/6 background. The single knockout *sr-a-/-* and *cd36-/-* mice were derived from double knockout *sr-a/cd36-/-* mice kindly provided by Dr Valérie Quesniaux (CNRS, Orléans, France). Animals were housed in the Institut Pasteur animal facilities under specific pathogen-free conditions. All mice stayed at least 1 week in the same room before experimentation. Protocols, performed in compliance with the NIH Animal Welfare Insurance #A5476-01 issued on 02/07/2007, and the French and European Union guidelines and regulations on handling, care and protection of Laboratory Animals (http://ec.europa.eu/environment/chemicals/lab_animals/home_en.htm). Protocols were approved by the veterinary staff of Institut Pasteur animal care and Ile-de-France Paris 1 ethical committee (authorization n°: 2011-0002).

### Bacteria, bacterial growth and infection

*S. aureus* strain HG001 [32] a *rsbU* ^+^ variant of strain NCTC 8325, a genetically tractable, clinically relevant non-mutagenized strain was used. HG001 is repaired for the rsbU allele, which restores the activity of the alternative sigma factor Sigma B. This strain has never been subjected to mutagenesis or UV treatment. It is methicillin sensitive and contains three resident prophages, including the one carrying the staphylokinase gene. The strain makes efficient biofilms and is highly virulent, with a pathogenicity reportedly similar to that of the USA300 strain. Bacteria were isolated on trypto-caseine soja agar medium (TSA) (BD Biosciences, Franklin Lakes, NJ, USA). One colony was pre-cultured overnight in trypto-caseine soja broth (TSB - BD Biosciences). The next day, bacteria were cultured in TSB at 37°C until they reached the exponential growth phase. Bacteria were then centrifuged (10 minat 4,000 g and 4°C), washed once with saline (Fresenius Kabi, Bad Homburg, Germany) and put in suspension at the required concentration. For intranasal infection, mice were anesthetized with an intraperitoneal injection of 100 µl of PBS containing 0.2% of xylazine (Rompun; Bayer; Leverkusen, Germany) and 1% of ketamine (Imalgen 1000; Merial; Lyon, France). They were then instilled intranasally with 20 µl of the bacterial suspension (containing 10^9^ bacteria) at day 0. After instillation, mice were sustained in a vertical position during 30 sec to allow the bacteria to go down the broncho-alveolar tree. For the peritoneal model of infection, mice were injected i.p. with 5×10^7^ CFU/g of body weight.

### Broncho-alveolar lavages (BAL) and peritoneal lavages (PL) fluids and plasma

Mice were sacrificed post-infection with pentobarbital (CEVA santé animale; Libourne, France) diluted 1∶5 with saline. Lungs were flushed with 500 µl of saline to obtain BAL fluids. Cells were isolated from the peritoneal cavity by washing with ice-cold RPMI 1640 (Glutamax; Lonza, Basel, Switzerland). Blood was taken by intra-cardiac puncture with a 1 ml syringe containing 100 µl of heparin (100 U.I/ml) (Sanofi-Synthelabo, Le Plessis Robinson, France). Blood, BAL and PL fluids were plated at various dilutions on TSA Petri dishes for colony forming unit (CFU) counting, the remaining volume was centrifuged at 300 g for 10 min at 4°C. The leukocyte pellet from BAL and PL fluids was analyzed by flow cytometry. Supernatants and plasma were filtered (0.22 µm) using 160 Spin X columns (CoStar, Corning Lifesciences, Lowell, MA, USA) and kept at −20°C for cytokine dosage.

### Cell counting and flow cytometry

Leukocytes in BAL and PL fluids were resuspended in 180 µl MACS buffer (PBS containing 0.5% FCS and 2 mM EDTA) with 20 µl mouse Fc block reagent (Myltenyi Biotec, Auburn, CA), incubated for 10 min at 4°C and divided in two. One half was incubated for 30 min on ice in MACS buffer with an anti-F4/80 antibody coupled to pacific blue (Biolegend, San Diego, CA) and an anti-Gr1 antibody coupled to PE (Miltenyi Biotec). The other half was incubated with a rat IgG2b-pacific blue (Biolegend) and a rat IgG2b-PE (AbD Serotec, Düsseldorf, Germany) as isotype controls. Cells were then washed and resuspended in 300 µL of MACS buffer. Data were acquired on 30 µl of the cell suspension using a MACSQuant flow cytometer that allows absolute cell counting. Neutrophil and macrophage counts were determined by gating on Gr1-positive/F4/80-negative or F4/80-positive cells, respectively, using the MACSQuantify software and taking into account the total volume of the samples.

### Histological analysis

Lungs from mice infected intranasally were removed and immediately fixed in 10% neutral-buffered formalin. After 48 h of fixation, lung samples were embedded in paraffin; 4 micrometer sections were cut and stained with hematoxylin and eosin, or Gram to highlight tissue lesions or to detect bacteria, respectively. A double blind histological analysis was performed.

### ELISA

IL-1β, IL-6, IL-10, TNFα and KC concentrations in BAL, PL fluids and plasma were determined by ELISA as specified by the manufacturer (DuoSet, R&D systems, Minneapolis, MN).

### Statistical analysis

Data were expressed as individual values and median. Statistical analysis was performed with the Graph Pad Prism software using Mann-Whitney signed rank test. The mortality was analyzed using the Kaplan-Meier test. A *P* value below 0.05 was considered to be significant.

## Results

### The susceptibility to *S. aureus* depends on the site of infection

In the light of the heterogeneity of the bacterial strains used so far in the literature to study *S. aureus*-host interaction, we decided to perform our study with a well-characterized strain (HG001) [32].

**Figure 1:**
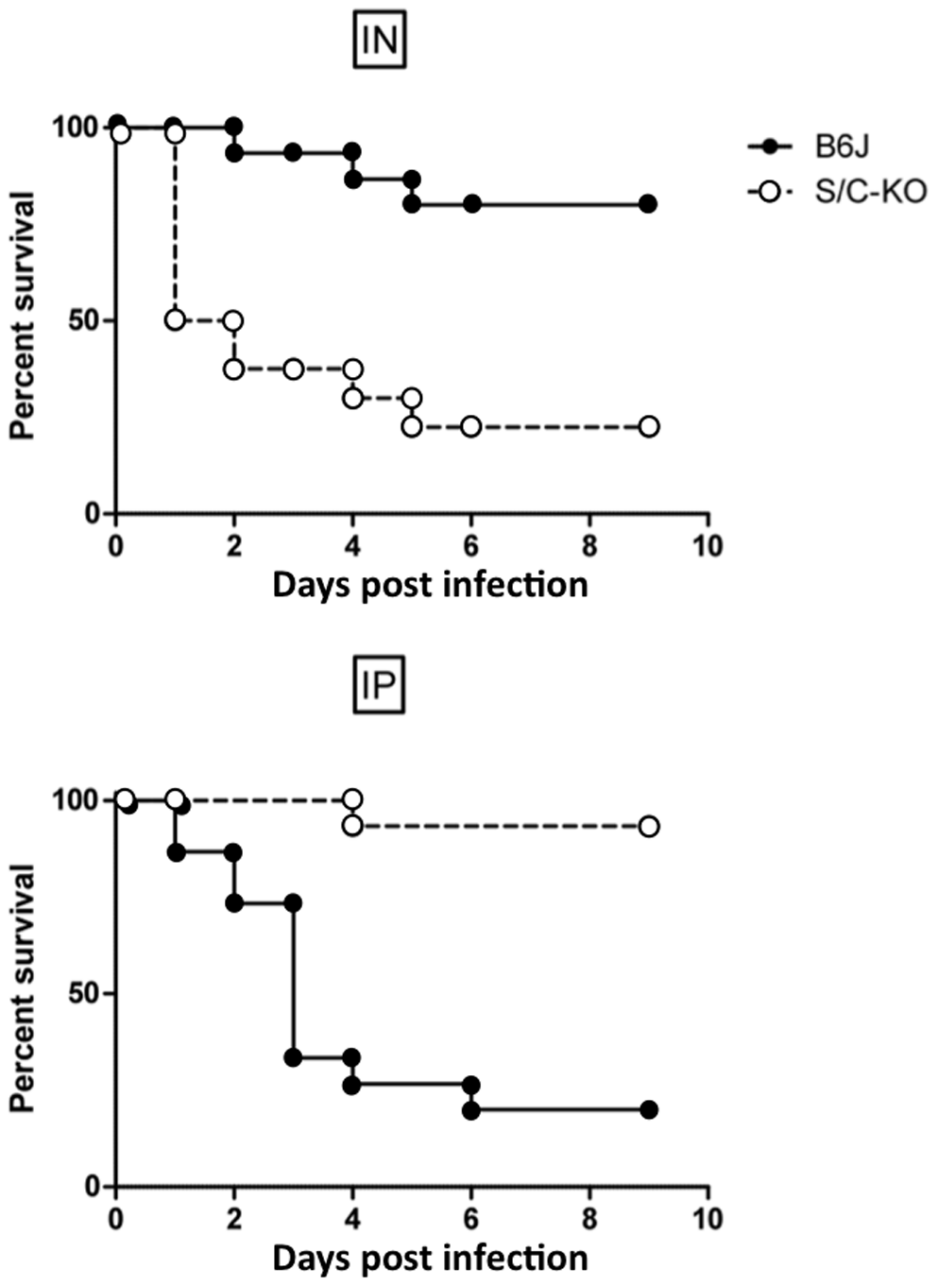
Survival curves. Survival curves of C57BL/6 (B6J) and double SR-A/CD36 knockout (S/C-KO) mice after a pulmonary infection following an intranasal inoculation (IN) of 10^9^ CFU of *S. aureus* (upper panel) or a peritoneal injection (IP) of 5×10^7^ CFU of *S. aureus*/g of mice (lower panel). The results have been acquired with n = 14 B6/J and n = 14 S/C-KO for lung infection, and n = 15 B6/J and n = 15 S/C-KO for peritoneal infection.

The mortality of wild type C57BL/6 (B6J) and *sr-a/cd36* double KO mice (S/C-KO) was followed after intra-peritoneal (i.p.) or intranasal (i.n.) infection. As shown in Figure 1, B6J mice were resistant to i.n. infection, whereas the knockout mice were highly sensitive. Interestingly, the results were completely mirrored when the infection was i.p.; in this case B6J mice were highly susceptible to infection as 80% of them succumbed to it, whereas knockout mice were resistant. Using single knockout *sr-A -/- and cd36 -/-* mice, we observed very similar results (Figure S1). Even if the absence of one receptor was sufficient to observe a different phenotype in terms of mortality, we preferred to investigate double knockout animals to have both types of scavenger receptors absent and avoid that one of them substitute the absence of the other [31], preventing us to have a distinct phenotype. Consequently, the rest of the study was performed with double knockout mice. Interestingly, the deletion of two other innate immune receptors involved in *S. aureus* recognition, namely TLR2 and MARCO ended to a similar observation. Indeed, the deletion of MARCO or TLR2 was protective in peritoneal infection while in contrast their absence was detrimental after pulmonary infection with *S. aureus* (figure S2).

### Bacterial load and phagocytes counts in the two models of infection are different

**Figure 2:**
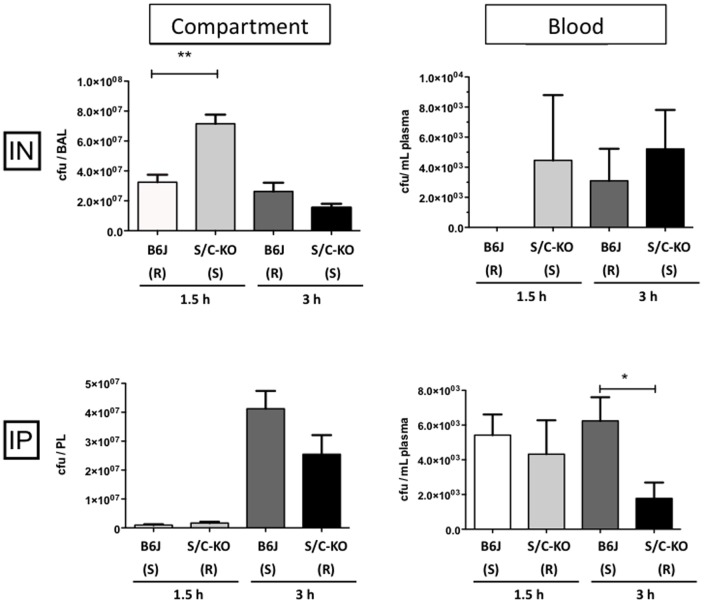
Bacterial counts in broncho-alveolar lavage (BAL) fluids (after intranasal inoculation of 10^9^ CFU of *S. aureus*), in peritoneal lavage (PL) fluids (after peritoneal injection of 5×10^7^ CFU of *S. aureus*/g of mice, and in blood for both types of infection. Samples from C57BL/6 (B6J) and double SR-A/CD36 knockout (S/C-KO) were harvested 1.5 h and 3 h after infection. (R) and (S) stand for resistant and susceptible mice depending on the route of infection. All data acquired after 1.5 h are the mean of n = 5 mice, data acquired after 3 h are the mean of 10, 10, 7 and 5 mice in the peritoneal cavity, the broncho-alveolar lavages, and in the blood (after intra-nasal infection and intraperitoneal infection), respectively. * *P*<0.05; ** *P*<0.01 between wild type and deficient mice.

To better characterize the difference observed for mortality between the strains depending on the route of infection, we analyzed the colony-forming units (CFU) locally and in the blood of infected animals. For the intranasal infection model, the susceptible mice (S/C-KO) had more CFU in the bronchoalveolar lavage (BAL) fluids soon after infection (1.5 hours) (Figure 2). Furthermore, for i.n. route the bacteria rarely spread in the blood: circulating colonies were recovered only in few mice suggesting that the infection remained local in contrast to the i.p. infection, which became rapidly systemic with higher amounts of CFU in the blood of wild type (susceptible) mice. In contrast no difference was seen within the peritoneal cavity.

**Figure 3:**
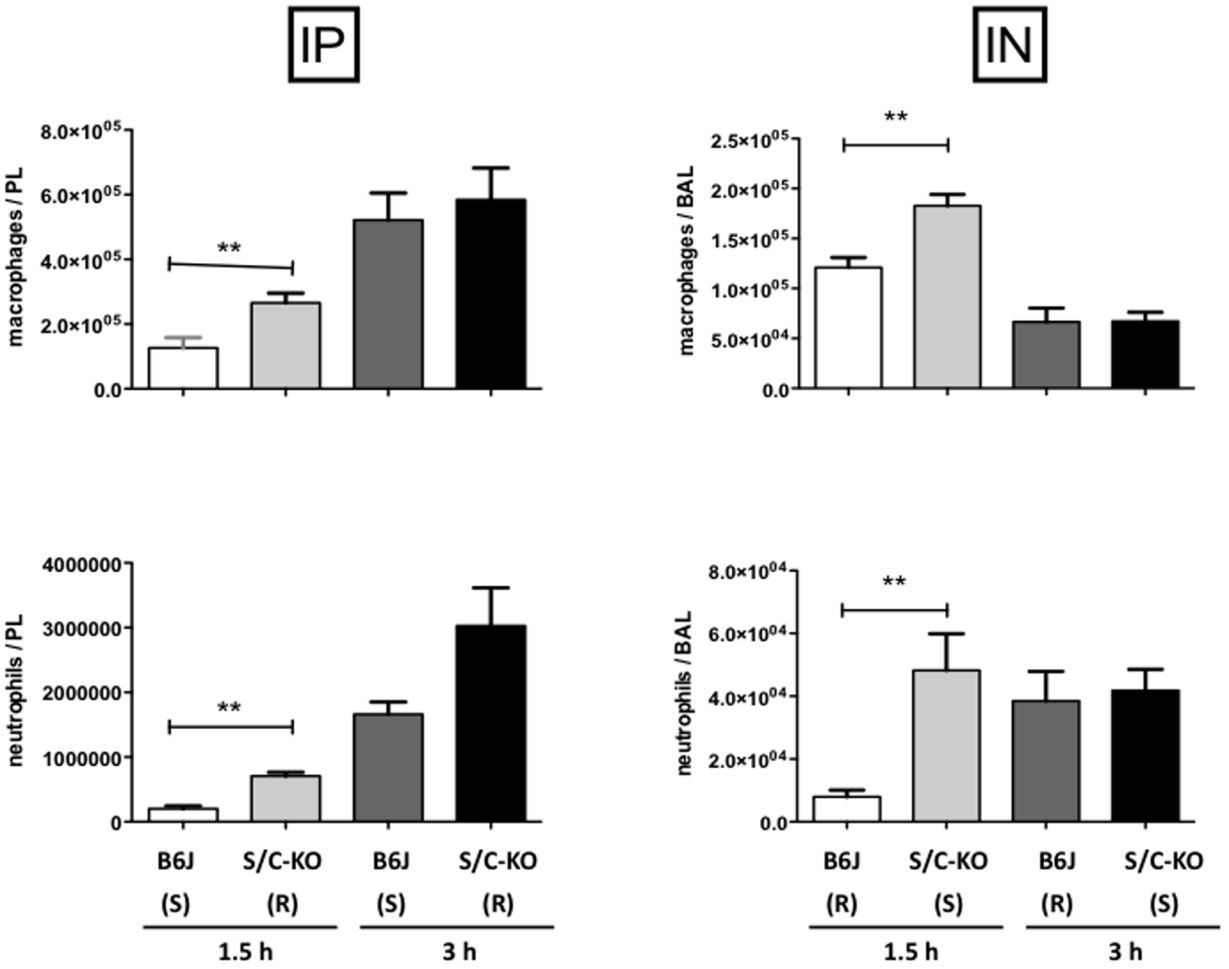
Macrophage and neutrophil counts in broncho-alveolar lavage (BAL) fluids (after intranasal inoculation of 10^9^ CFU of *S. aureus*), and in peritoneal lavage (PL) fluids (after peritoneal injection of 5×10^7^ CFU of *S. aureus*/g of mice. Samples from C57BL/6 (B6J) and double SR-A/CD36 knockout (S/C-KO) were harvested 1.5 h and 3 h after infection. (R) and (S) stand for resistant and susceptible mice depending on the route of infection. All data acquired after 1.5 h are the mean of n = 5 mice, and all data acquired after 3 h are the mean of 10 mice. ** *P*<0.01 between wild type and deficient mice.

There was no difference between B6J and S/C-KO mice in terms of number of resident macrophages in the peritoneal cavity or the broncho-alveolar space before infection (data not shown). Significantly more macrophages and neutrophils were found at 1.5 hours for resistant mice (S/C-KO) after i.p. infection within the peritoneal cavity (Figure 3). This difference was not significant anymore at 3 hours, even if a trend was observed for more phagocytes in the case of resistant mice. Surprisingly, after i.n. infection, an early increase in the number of macrophages and neutrophils was also found for S/C-KO mice, within the broncho-alveolar lavages despite their higher susceptibility to *S. aureus* in this case.

### Lung lesions evaluated by histology

Histology was performed to see if the differences seen in terms of mortality or bacterial load were linked to a difference in terms of lesions. Histological analysis revealed similar lesions, in nature and severity, in control and S/C-KO mice for the lungs after 12 hours post i.n. infection, *i.e.* a multifocal to coalescing bronchopneumonia, each focus measuring from 100 to 800 µm in diameter, and characterized by infiltrates of neutrophils (a high proportion of these neutrophils being fragmented and karyorrhectic) and macrophages, mostly centered around bronchi and bronchioles but also extending to alveoli. A high density of Gram-positive bacteria was observed in both groups. These results suggest that in the early phase of the infectious process the deficiency in SR-A and CD36 does not significantly modify the lesional inflammatory response (data not shown).

### Cytokine production for resistant and susceptible mice in i.p. versus i.n infection

**Figure 4:**
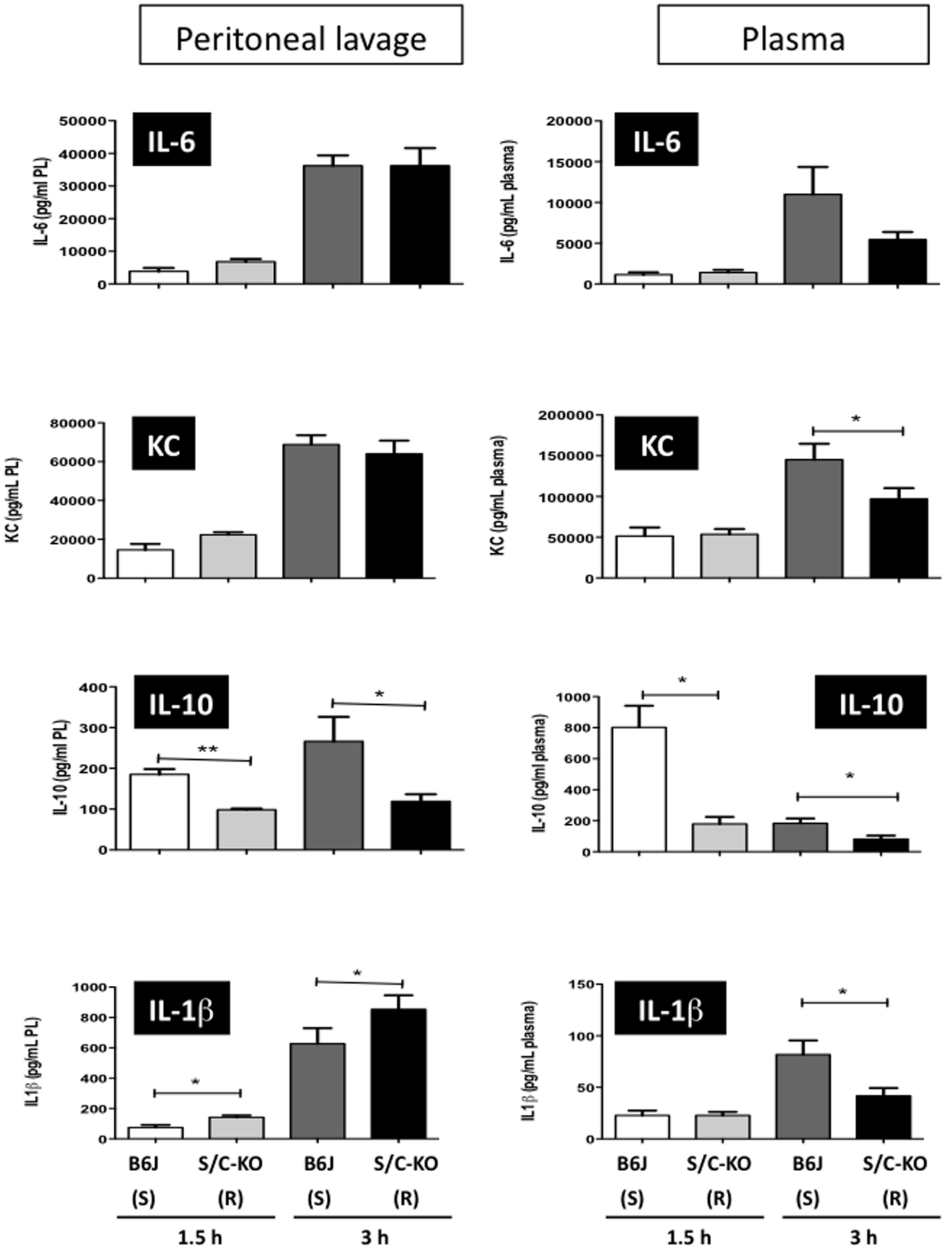
Cytokine levels (IL-6, KC, IL-10 and IL-1β) in peritoneal lavage (PL) fluids and in plasma after peritoneal injection of 5×10^7^ CFU of *S. aureus*/g of mice. Samples from C57BL/6 (B6J) and double SR-A/CD36 knockout (S/C-KO) were harvested 1.5 h and 3 h after infection. (R) and (S) stand for resistant and susceptible mice. All data acquired after 1.5 h are the mean of n = 5 mice, data acquired after 3 h are the mean of 10 and 5 mice in the peritoneal cavity and in the blood compartment, respectively. * *P*<0.05.

**Figure 5:**
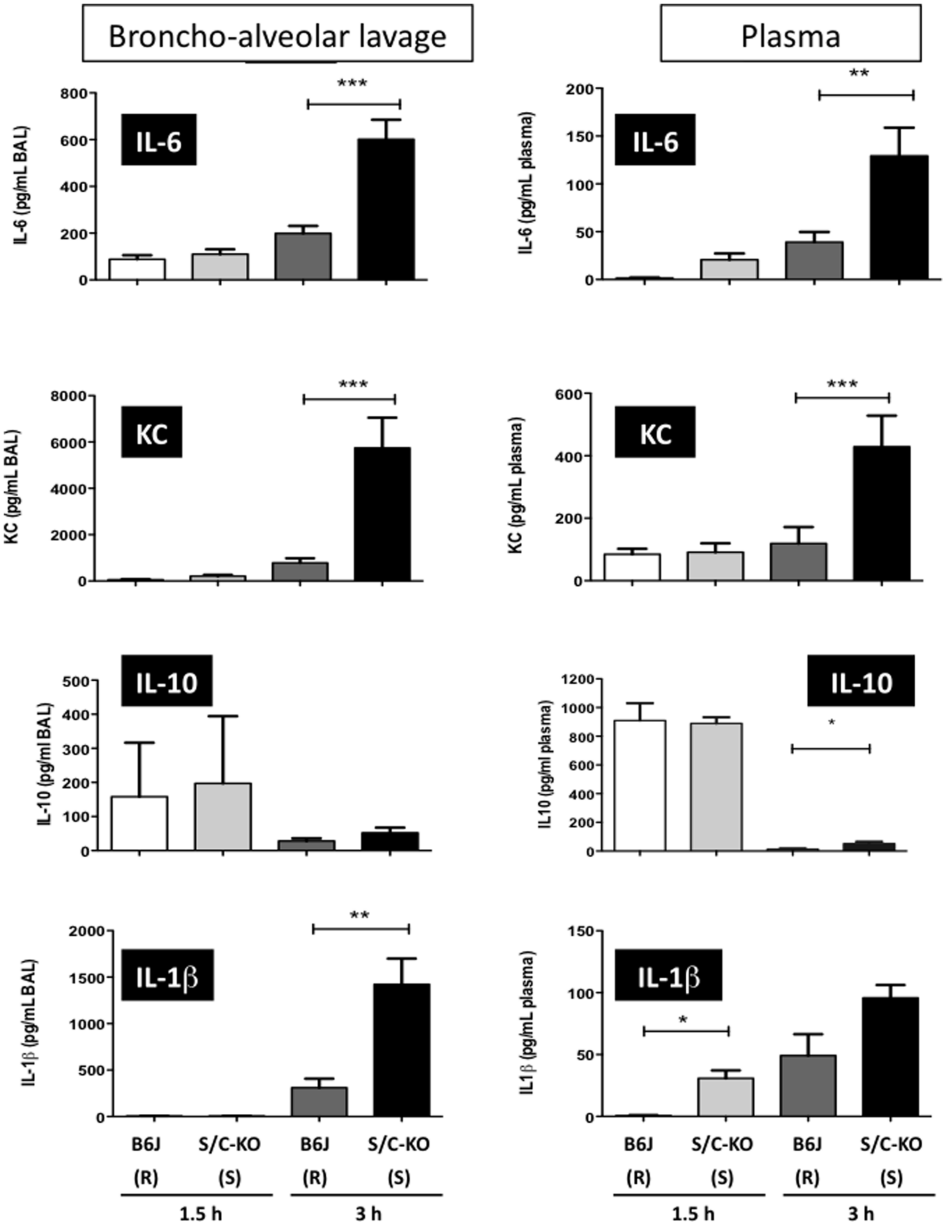
Cytokine levels (IL-6, KC, IL-10 and IL-1β) in broncho-alveolar lavage (BAL) fluids and in plasma after intranasal inoculation of 10^9^ CFU of *S. aureus*. Samples from C57BL/6 (B6J) and double SR-A/CD36 knockout (S/C-KO) were harvested 1.5 h and 3 h after infection. (R) and (S) stand for resistant and sensitive mice. All data acquired after 1.5 h are the mean of n = 5 mice, and all data acquired after 3 h are the mean of 10 mice. * *P*<0.05 ; ** *P*<0.01 ; *** *P*<0.001 between wild type and deficient mice.

Next we measured the presence of cytokines and chemokines (IL-1β, IL-6, IL-10, and KC) in the lavage fluids of the infected compartment and in the plasma. After i.p. infection (Figure 4), locally very few differences were seen between susceptible and resistant mice. Only IL-1ß was locally present at higher concentrations for resistant mice (S/C-KO), at both time points. Interestingly, in the same time the levels of the anti-inflammatory cytokine IL-10 were significantly lower. For the other cytokines found in peritoneal lavages, comparable levels were detected in both groups (including TNFα, data not shown). In the plasma, differences were also noted. Less IL-1β and KC were found at 3 hours for the resistant mice (S/C-KO), as well as less IL-10 both at 1.5 and 3 hours post-infection. No difference was seen between susceptible and resistant mice for plasmatic TNFα (data not shown). For the i.n. infection, cytokine levels were very low locally at 1.5 hours but increased levels of IL-6, KC and IL-1β were found at 3 hours for susceptible mice (S/C-KO) (Figure 5). IL-10 was detected at 3 hours, but without significant difference between the two groups. Similarly, TNFα was present in BAL fluids but no difference was seen between susceptible and resistant mice (data not shown). The same situation was observed in the plasma, where more IL-1β was found at the early time point. Of note are the high early levels of IL-10 in both sensitive and resistant mice. At 3 hours, IL-6, KC and IL-10 were significantly increased for susceptible mice (S/C-KO). The levels of IL-1β in the plasma were comparable to those of the i.p. infection, but those of KC and IL-6 were much lower, probably because as seen in Figure 2 few CFU spread in the bloodstream in this model. TNFα levels were very low in the plasma and most of the time below the detection limit (data not shown).

## Discussion

The aim of this study was to revisit the resistance of mice to *S. aureus* infection, evaluating the impact of the route of infection and the role of scavenger receptors SR-A and CD36 in the resistance or susceptibility. Our objective was to reconcile some contradictory data found in the literature about the susceptibility of mice deficient for innate immune receptors. This contradiction may be due to the use of more or less virulent *S. aureus* strains, including clinical isolates, not fully characterized for the expression of virulence factors. To overcome this difficulty, we used a well-characterized strain of *S. aureus* (HG001) [32], which expresses virulence factors known to be important for *S. aureus* pathogenicity. The second source of dissonance in the literature may be due to the site of infection. We thus chose to compare two compartments: the peritoneal cavity and the lungs, which are the most frequent sites of infection *with S. aureus* for patients admitted in ICU. These two models give also the advantage to compare a systemic (i.p.) to a more localized infection (i.n.), as assessed by the very low spreading of CFU in the blood in the latest model.

Comparing the S/C-KO mice with their WT counterpart (B6J) we found interestingly that mice susceptible to i.p. infection were resistant to i.n. infection and vice versa. S/C-KO mice were resistant to i.p. infection. They had less CFU spreading in the blood and higher numbers of macrophages and neutrophils were found locally in the early time. This observation is in agreement with the fact that both cell types contribute to the anti-infectious process against *S. aureus* thanks to their phagocytic activity [34]–[38]. The involvement of CD36 [17], [18] and SR-A [39], [40] in *S. aureus* phagocytosis has been clearly established. The reduced susceptibility of S/C-KO mice to i.p. infection may be due to the reduced spreading of the bacteria into the bloodstream as compared to WT animals. It results that the systemic inflammatory response, illustrated by cytokines, is reduced in S/C-KO mice as compared to B6J mice. It is well known that high levels of pro-inflammatory cytokines in the blood create a systemic inflammation that may lead to organ dysfunction and death [41]. Regarding the intra-peritoneal infection, the striking difference is the higher level of both local and systemic IL-10 in the susceptible mice (B6J) and the higher level of IL-1β in the peritoneal cavity of resistant mice (S/C-KO). Indeed, on one hand IL-10 is known to be an immunosuppressive cytokine than can alter the immune defense [42]–[44], and on the other hand IL-1β and the inflammasome required for its maturation both contribute to the protection against *S. aureus* infection [45], [46].

Interestingly, the same S/C-KO mouse strain was highly susceptible to i.n. infection by *S. aureus*. They had more CFU in the lungs, but paradoxically they had also more macrophages and neutrophils. After intra-nasal delivery of *S. aureus* and pulmonary infection, susceptible S/C-KO mice displayed also higher levels of cytokines, both locally and systemically. This observation is reminiscent of the detrimental role of cytokines of which excessive amounts can lead to shock and multiple organ failure. For example, higher plasma levels of cytokines were found in the susceptible mouse strain (A/J) as compared to a more resistant one (C57BL/6) after systemic infection with *S. aureus* [35]. Furthermore, a protective therapeutic approach against lethal *S. aureus* peritoneal infection in mice was associated with a reduced level of inflammatory cytokines [47]. In our model, the absence of SR-A and CD36 induces a higher recruitment of phagocytes and a stronger inflammatory response in the lungs, which probably leads to the dysfunction of this organ and death. For ICU patients, lungs are most of the time the first organ showing failure. The phenomenon observed for S/C-KO mice is very fast, as most of the animals die within 24 hours, before creating visible lesions in the lungs.

Most importantly, our data demonstrate that depending upon the compartment, similar receptors can play contrasting role. In the case of *S. aureus* infection, such beneficial or detrimental role has already been attributed to plasminogen [48] and to gamma-interferon [49]. In the later case, the report demonstrated that the mechanisms of host defense are greatly influenced by their tissue environment since this cytokine had a positive effect to control systemic sepsis but a negative role for *S. aureus*-associated arthritis. Similarly, IL-10 was protective in *Francisella tularensis* pulmonary infection but deleterious after cutaneous inoculation [50]. In addition, the cells are greatly influenced by their local microenvironment. For example, macrophages are extremely different from one compartment to another and their capacity to respond to bacteria or bacterial products differs from one site to another as shown for intestinal, peritoneal, alveolar, spleen, microglial macrophages and monocytes [12], [51]–[53].

However, we failed to find any significant differences in terms of cytokine production when comparing the *in vitro* capacity of peritoneal macrophages of wild type mice and S/C-KO mice to produce IL-1β, IL-6, TNF and KC in response to LPS and heat-killed *S. aureus* RN1HG (data not shown). A similar observation was made for alveolar macrophages, and for the phagocytic activity of both types of macrophages from wild type and S/C-KO mice (data not shown).

Regarding cell surface receptors involved in the recognition of pathogen-associated molecular patterns, their role may vary depending on the tissue. This has been shown for TLR2 of which the presence within the kidney influences *S. aureus* clearance, whereas its expression in lungs, liver or spleen does not affect the bacterial clearance [54]. A similar observation in a peritonitis model was reported for TLR4 of which the expression on myeloid cells was protective while its expression on hepatocytes was deleterious [55].

In conclusion, our report demonstrates that similar cell surface innate immune receptors (CD36, SR-A, MARCO and TLR2) play different roles against *S. aureus* infection, depending on the site of infection. It enlightens the concept of compartmentalization of the innate immune response of which mechanisms can be completely different depending on the tissue/organ where it occurs [56]. A careful analysis, keeping in mind this concept of compartmentalization, should avoid dogmatic statements about the role of the different actors involved in anti-infectious processes of the host.

## Supporting Information

Figure S1 **Mortality of single knockout mice after infection with** ***S. aureus*** **is similar to that of double deficient mice.** (A) Mortality was followed after intranasal (IN) or (B) intra-peritoneal (IP) infection. WT C57BL/6J (black) and *cd36*-/- (blue) and *sr-a*-/- (red) were compared. The results were acquired with n = 11 and n = 7 C57BL/6J, n = 8 and n = 5 *cd36* -/-, and n = 8 and n = 6 *sr-a* -/- mice, for peritoneal and pulmonary infections, respectively.
(TIF)

Figure S2 **Survival curves of C57BL/6J,** ***marco*** **-/- and** ***tlr2*** **-/- mice after either a pulmonary infection following an intranasal (i.n.) inoculation of 10^9^ CFU of** ***S. aureus*** **(left figures) or a peritoneal injection (i.p.) of 5×10^7^ CFU of** ***S. aureus*** **/g of mice (right figures).** The results were acquired with n = 10 and n = 5 C57BL/6J, n = 8 and n = 5 *marco* -/-, and n = 10 and n = 17 C57BL/6J n = 9 and n = 10 *tlr2* -/-, for peritoneal and pulmonary infections, respectively.
(TIF)
