# Supplementary figures and images for: Protective or Deleterious Role of Scavenger Receptors SR-A and CD36 on Host Resistance to Staphylococcus aureus Depends on the Site of Infection
Source: PLoS One. 2014 Jan 31;9(1):e87927. doi: 10.1371/journal.pone.0087927 (PMC3909292; doi:10.1371/journal.pone.0087927)

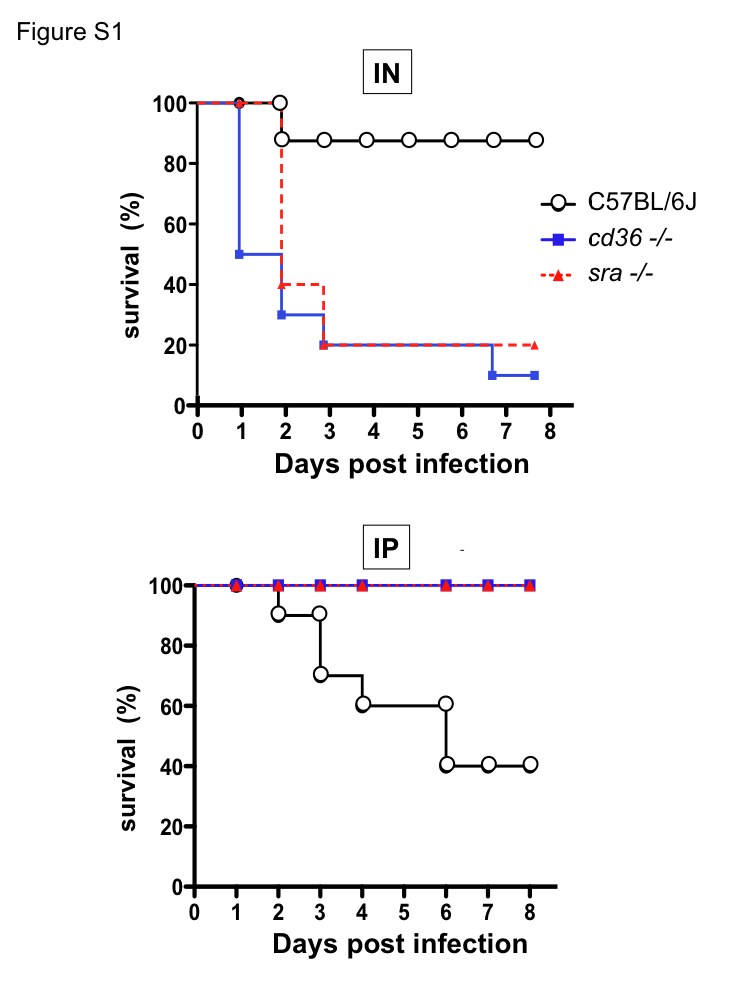

Supplement: Figure S1 — Mortality of single knockout mice after infection with S. aureus is similar to that of double deficient mice. (A) Mortality was followed after intranasal (IN) or (B) intra-peritoneal (IP) infection. WT C57BL/6J (black) and cd36-/- (blue) and sr-a-/- (red) were compared. The results were acquired with n = 11 and n = 7 C57BL/6J, n = 8 and n = 5 cd36 -/-, and n = 8 and n = 6 sr-a -/- mice, for peritoneal and pulmonary infections, respectively. (TIF) [file pone.0087927.s001.tif]

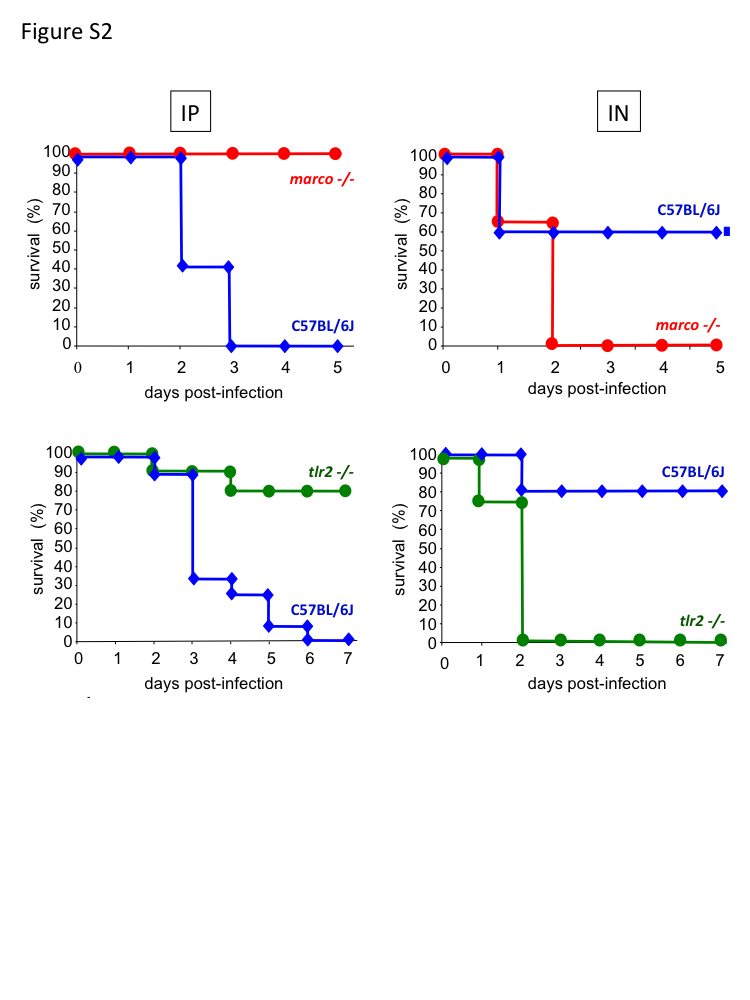

Supplement: Figure S2 — Survival curves of C57BL/6J, marco -/- and tlr2 -/- mice after either a pulmonary infection following an intranasal (i.n.) inoculation of 109 CFU of S. aureus (left figures) or a peritoneal injection (i.p.) of 5×10^7^ CFU of S. aureus /g of mice (right figures). The results were acquired with n = 10 and n = 5 C57BL/6J, n = 8 and n = 5 marco -/-, and n = 10 and n = 17 C57BL/6J n = 9 and n = 10 tlr2 -/-, for peritoneal and pulmonary infections, respectively. (TIF) [file pone.0087927.s002.tif]
